# Item

**Published:** 1901-10

**Authors:** 


### ITEM.

THE examination of applicants for appointment as assistant surgeon in the army has been resumed in Washington and San Francisco; the army medical boards convened in those cities will remain in session as long as there are candidates to be examined. Seventy-six vacancies in the medical department still remain to be filled, and as it is desired by the military authorities that the department be filled up to its full legal limit as early as practicable, all eligible applicants will be afforded opportunity for examination; those found qualified will be commissioned at an early date. Full information as to eligibility, nature and scope of examination, etc., may be obtained upon application to the Surgeon-General, U. S. Army, Washington, D. C.

---

### EXPLANATION.

MANY of the usual features of the JOURNAL have been forced to give way this month to the material relating to the President's case. We feel sure our readers, subscribers and advertisers, will recognise the paramount importance of this subject, and acquiesce in the precedence it is accorded.

